# Impact of Calcium Phosphate Product on Acute Kidney Injury and Mortality: A Retrospective Cohort Study

**DOI:** 10.7759/cureus.64861

**Published:** 2024-07-18

**Authors:** Ratchapong Jetanapirom, Ussanee Boonsrirat, Sarayut L Geater, Rattana Leelawattana, Atthaphong Phongphithakchai

**Affiliations:** 1 Internal Medicine, Prince of Songkla University, Songkhla, THA

**Keywords:** nephrology, mortality, hospitalized patients, acute kidney injury, serum calcium phosphate

## Abstract

Purpose: This study aims to assess the association between admission-corrected serum calcium phosphate (CaPO_4_) levels and the risks of in-hospital acute kidney injury (AKI) and mortality, hypothesizing a dose-dependent relationship between serum CaPO_4_ concentrations and the likelihood of developing AKI.

Methods: This large retrospective cohort study analyzed hospitalized adult patients who had serum calcium, phosphate, and albumin levels measured within 24 hours of admission between January 2014 and December 2018. Piecewise regression was employed to identify the optimal CaPO_4_ cutoff values for predicting in-hospital AKI and mortality. Subsequently, the odds ratios (ORs) and 95% confidence intervals (CIs) were calculated to assess the risks of in-hospital AKI and mortality associated with these cutoff values.

Results: A total of 2,116 patients were included in the study. The incidence rates of AKI for patients with CaPO_4_ levels ≤27 and >27 mg^2^/dL^2^ were 9.6% and 10.9%, respectively. The bilinear association pattern revealed the lowest risk of AKI at a CaPO_4_ level of 27 mg^2^/dL^2^. Piecewise regression analysis showed that each 1 mg^2^/dL^2^ increase in CaPO_4_ level above the 27 mg^2^/dL^2^ cutoff was associated with increased risks of in-hospital AKI and mortality, with OR of 1.048 (95% CI: 1.030-1.065) and 1.048 (95% CI: 1.032-1.065), respectively.

Conclusion: Our findings indicate a critical relationship between elevated serum CaPO_4_ levels and increased risks of in-hospital AKI and mortality, with a notable cutoff at CaPO_4_ >27 mg^2^/dL^2^.

## Introduction

Acute kidney injury (AKI) is a common and life-threatening consequence of acute illnesses and is associated with poor clinical outcomes, even with slight reductions in kidney function [1]. Approximately 20% of hospitalized patients experience AKI, which contributes to more than 1.7 million deaths annually [2]. Several studies have reported data on biomarkers for early diagnosis and have identified various preventable risk factors associated with AKI. These studies highlight critical opportunities for improving management and patient outcomes [3,4].

Serum calcium phosphate (CaPO_4_) is proposed as a strong risk factor for AKI [5-9]. Previous studies have elucidated specific risk factors, such as the use of oral sodium phosphate for bowel preparation and both oral and intravenous phosphate supplements. These are linked to acute phosphate nephropathy and subsequent AKI [5-7]. Notably, elevated CaPO_4_ levels in the loop of Henle are associated with a higher propensity for CaPO_4_ crystallization, thus increasing the risk of AKI [8]. Additionally, the serum CaPO_4_ product independently predicts AKI during hospital stays [9]. Serum CaPO_4_ levels are also well-documented as predictors of adverse outcomes, which include secondary hyperparathyroidism, vascular calcifications, and significant cardiovascular events, leading to increased mortality among patients with chronic kidney disease (CKD), end-stage renal disease (ESRD), and cardiovascular diseases [10-12]. These findings highlight the impact of serum CaPO_4_, showing its role in causing AKI and increasing the risk of hospital mortality and long-term complications [13,14].

However, current data on the precise impact of serum CaPO_4_ levels on in-hospital AKI and mortality are limited. Thus, we aim to evaluate the risk of in-hospital AKI in adults upon admission based on the patient’s serum CaPO_4_ levels. We hypothesized that higher serum CaPO_4_ levels will be associated with an increased risk of in-hospital AKI.

## Materials and methods

Study population

This single-center retrospective cohort study was conducted at a tertiary hospital in Southern Thailand. We included adult patients admitted to the internal medicine ward from January 2014 through December 2018. Eligible patients were required to have measurements of serum calcium, phosphate, and albumin within 24 hours of admission. Inclusion criteria involved one of the following: 1) a baseline serum creatinine (Cr) measurement within three months prior to admission, a serum Cr measurement within 24 hours of admission, and a repeated serum Cr measurement within one week of admission; 2) a baseline serum Cr within three months prior to admission, a serum Cr within 24 hours of admission, but no repeated serum Cr within one week of admission; 3) a serum Cr within 24 hours of admission and another within one week of admission, with no evidence of CKD. We excluded patients with ESRD, those undergoing renal replacement therapy, or those with a history of kidney transplantation. Additionally, patients who met the Kidney Disease Improving Global Outcomes (KDIGO) 2012 criteria for AKI at the time of admission were excluded. This study was approved by the Ethics Committee of the Prince of Songkla University (REC. 62-175-14-4).

Data collection and definitions

Data collection for this study was conducted using the institutional electronic medical record system. We gathered demographic data, clinical characteristics, admission notes, progress notes, discharge summaries, and laboratory data. The initial measurements of serum calcium, phosphate, and albumin within 24 hours of hospital admission were defined as the admission levels. Corrected serum CaPO_4_ levels were calculated using the equation: (serum calcium+0.8×(4-serum albumin))×serum phosphate [15,16]. The estimated glomerular filtration rate (eGFR) was calculated using the chronic kidney disease epidemiology collaboration (CKD-EPI) equation [17]. Information on the use of drugs, exposure to radiocontrast agents, mechanical ventilation, and vasopressor support were also collected. In-hospital AKI was defined by a sudden decline in kidney function during a hospital stay, characterized by an increase in serum Cr by 0.3 mg/dL or more within 48 hours or a rise to 1.5 times the baseline value within the previous seven days according to KDIGO guidelines [1,18].

The primary outcome is to investigate the relationship between corrected serum CaPO_4_ levels at admission and the incidence of in-hospital AKI. The secondary outcome is the relationship between admission-corrected serum CaPO_4_ levels and in-hospital mortality.

Statistical analysis

Descriptive statistics are presented as counts and percentages for categorical variables and as means ± SD for normally distributed data. Categorical variables were compared using Chi-square tests, while continuous variables were analyzed using t-tests or Mann-Whitney U tests, depending on their distribution. To explore the relationship between corrected serum CaPO_4_ levels and in-hospital AKI, piecewise regression analysis was employed to identify the optimal cutoff value that best fits the association, as indicated by the lowest Akaike’s Information Criterion (AIC) and Bayesian Information Criterion (BIC). A multivariable logistic regression model was used to assess factors associated with in-hospital AKI and in-hospital mortality. Models were adjusted for age, sex, race, underlying diseases, the use of medications, vasopressor usage, and receiving mechanical ventilation. Estimates were presented as odds ratios (ORs) with 95% confidence intervals (CIs). Statistical significance was established at a P-value of <0.05. Data analysis was conducted using Stata statistical software (version 17, StataCorp LLC, Texas, USA).

## Results

Study population 

**Figure 1:**
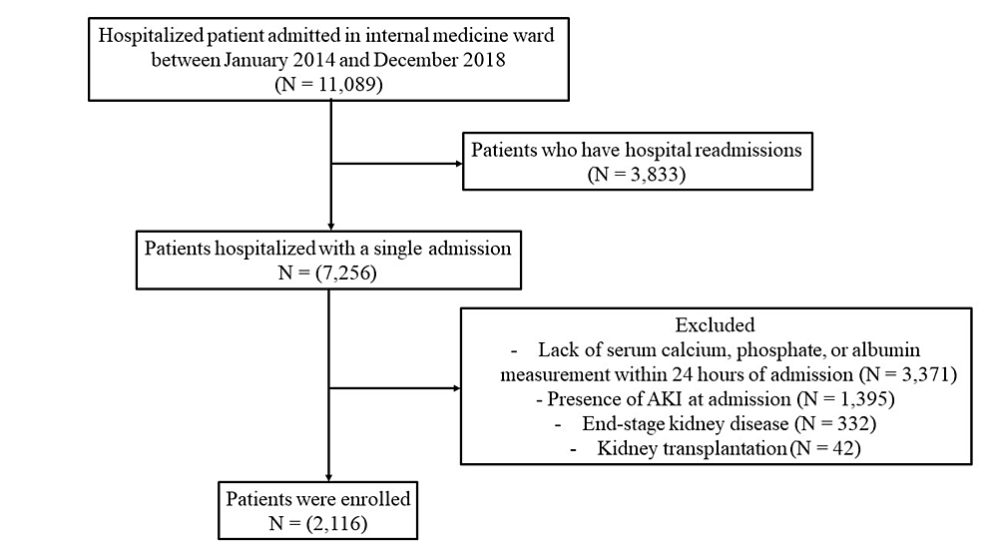
Flow chart of the study population

A total of 11,089 patients hospitalized between 2014 and 2018 were initially identified. Of these, 3,833 were excluded due to hospital readmissions. An additional 5,140 patients were excluded for one or more of the following reasons: lack of serum calcium, phosphate, or albumin measurements within 24 hours of admission; presence of AKI at admission; ESRD; or previous kidney transplantation, as shown in Figure 1. After applying these exclusion criteria, 2,116 patients remained eligible and were enrolled in the study. The baseline characteristics of these 2,116 patients are detailed in Table 1. Overall, 968 (45.7%) of the study population were female, with a mean age of 59.5±18.7 years. The most common comorbidities were hypertension, followed by diabetes. Most of the patients, up to 38.3%, received radiocontrast. The mean baseline Cr and serum Cr levels at admission were 1.0±0.6 mg/dL and 0.9±0.5 mg/dL, respectively. The mean-corrected CaPO_4_ level among the entire cohort was 31.3±9.5 mg^2^/dL^2^. Patients were divided into two groups based on their admission-corrected serum CaPO_4_ levels: Group 1 (≤27 mg^2^/dL^2^) and Group 2 (>27 mg^2^/dL^2^). Patients with CaPO_4_ ≤27 were significantly older and had higher incidences of chronic lung disease, chronic liver disease, and greater use of vitamin D supplements.

**Table 1:** Baseline characteristics and clinical outcomes Continuous data are demonstrated as mean ± SD; categorical data are demonstrated as count (percentage). Chi-square tests, t-tests, and Mann-Whitney U tests were performed. AKI, acute kidney injury; CaPO_4_, calcium phosphate; COPD, chronic obstructive pulmonary disease; Cr, creatinine; ACEI, angiotensin-converting enzyme inhibitor; ARB, angiotensin receptor blocker; NSAID, non-steroidal anti-inflammatory drug

	CaPO_4_ ≤27 mg^2^/dL^2^	CaPO_4_ >27 mg^2^/dL^2^	Total	P-value
	(N=659)	(N=1,457)	(N=2,116)	
Demographics				
Age, years	61.2±18.2	58.8±18.8	59.5±18.7	0.005
Female sex	293 (44.5%)	675 (46.3%)	968 (45.7%)	0.425
Comorbidities				
Hypertension	150 (22.8%)	329 (22.6%)	479 (22.6%)	0.927
Diabetes	132 (20.0%)	249 (17.1%)	381 (18.0%)	0.105
Coronary artery disease	55 (8.3%)	135 (9.3%)	190 (9.0%)	0.488
Stroke	73 (11.1%)	137 (9.4%)	210 (9.9%)	0.235
Asthma and COPD	48 (7.3%)	71 (4.9%)	119 (5.6%)	0.026
Chronic liver disease	49 (7.4%)	70 (4.8%)	119 (5.6%)	0.015
Medications				
ACEI/ARB	127 (19.3%)	252 (17.4%)	379 (18.0%)	0.290
Diuretics	219 (33.2%)	470 (32.3%)	689 (32.6%)	0.665
NSAIDs	53 (8.0%)	113 (7.8%)	166 (7.8%)	0.820
Aminoglycoside	7 (1.1%)	12 (0.8%)	19 (0.9%)	0.592
Macrolide	28 (4.3%)	50 (3.4%)	78 (3.7%)	0.355
Radiocontrast	254 (38.7%)	555 (38.2%)	809 (38.3%)	0.810
Calcium supplement	132 (20.0%)	277 (19.0%)	409 (19.3%)	0.588
Vitamin D supplement	53 (8.1%)	71 (4.9%)	124 (5.9%)	0.004
Vasopressors	99 (15.0%)	185 (12.7%)	284 (13.4%)	0.146
Mechanical ventilation	129 (19.6%)	236 (16.2%)	365 (17.3%)	0.058
Laboratory values				
Baseline serum Cr (mg/dL)	0.9±0.4	1.0±0.6	1.0±0.6	0.012
Serum Cr at admission (mg/dL)	0.9±0.4	1.0±0.6	0.9±0.5	<0.001
Clinical outcomes				
AKI	63 (9.6%)	159 (10.9%)	222 (10.5%)	0.347
In-hospital mortality	76 (11.5%)	155 (10.6%)	231 (10.9%)	0.541

Relationship between admission-corrected serum CaPO_4_ level and in-hospital AKI

**Figure 2:**
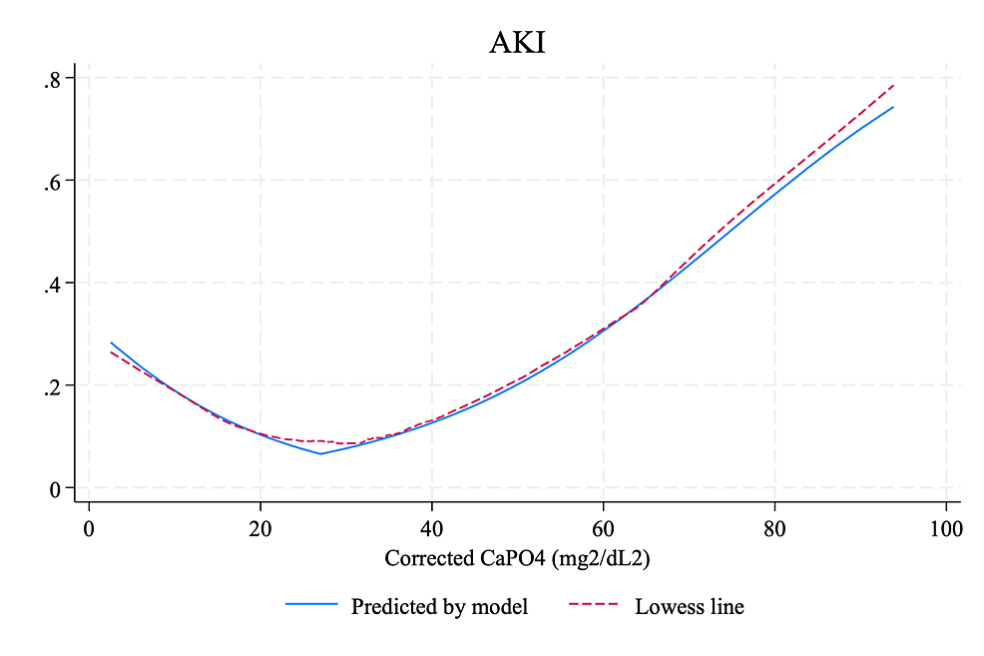
Piecewise regression analysis between admission-corrected serum CaPO4 and in-hospital AKI CaPO_4_, calcium phosphate

Among the 2,116 patients studied, 222 (10.5%) developed AKI within the first seven days of admission. No significant difference was observed in the incidence of in-hospital AKI between the two groups: 63 (9.6%) in Group 1 and 159 (10.9%) in Group 2 (P=0.347) (Table 1). Piecewise regression analysis identified 27 mg^2^/dL^2^ as the optimal cut point for CaPO_4_, showing the best fit with the lowest AIC and BIC. The model indicated that the probability of in-hospital AKI decreased as CaPO_4_ levels increased from 0 to 27. Beyond this point, the risk of AKI increased with higher CaPO_4_ levels (Figure 2). After adjusting for potential confounders, an increase of 1 mg^2^/dL^2^ in serum CaPO_4_ below 27 mg^2^/dL^2^ was associated with a 6.0% decrease in the likelihood of in-hospital AKI (OR: 0.940, 95% CI: 0.903-0.980). Conversely, for patients with initial CaPO_4_ levels above 27 mg^2^/dL^2^, each 1 mg^2^/dL^2^ increase in CaPO_4_ was associated with a 4.8% increase in the risk of developing in-hospital AKI (OR: 1.048, CI: 1.030-1.065) (Table 2).

**Table 2:** Piecewise regression analysis between admission-corrected serum CaPO4 and in-hospital AKI ^a^Adjusted for age, sex, race, underlying disease, the use of ACEI/ARB, diuretics, NSAID, aminoglycoside, macrolide, calcium supplement, vitamin D supplement, radiocontrast, vasopressor usage, and mechanical ventilator. The ORs presented are for the following parameters: CaPO_4_ ≤27 mg²/dL² and CaPO_4_ >27 mg²/dL². CaPO_4_, calcium phosphate; CI, confidence interval; OR, odd ratio; NSAID, non-steroidal anti-inflammatory drug; ACEI, angiotensin converting enzyme inhibitor; ARB, angiotensin receptor blocker

	Unadjusted			Adjusted^a^		
Variable	OR	95% CI	P-value	OR	95% CI	P-value
CaPO_4_ ≤27 mg^2^/dL^2^	0.932	0.900-0.968	<0.001	0.940	0.903-0.980	0.003
CaPO_4_ >27 mg^2^/dL^2^	1.057	1.042-1.073	<0.001	1.048	1.030-1.065	<0.001

Relationship between admission-corrected serum CaPO_4_ level and in-hospital mortality

**Figure 3:**
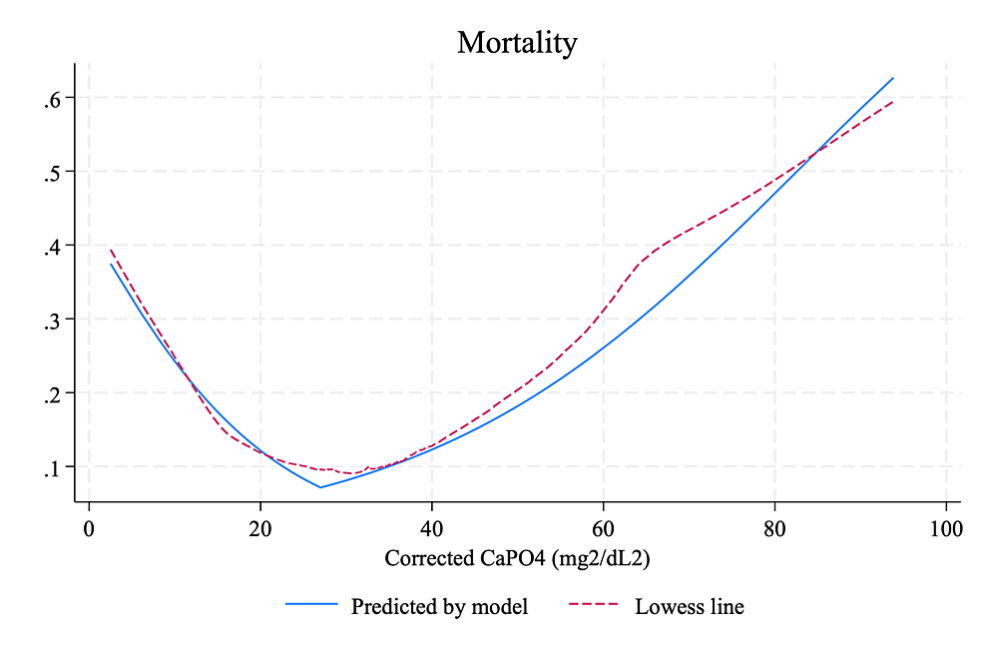
Piecewise regression analysis between admission-corrected serum CaPO4 and in-hospital mortality CaPO_4_, calcium phosphate

Of the 2,116 patients studied, 231 (10.9%) died while hospitalized. The mortality rates were comparable between the two groups: Group 1 had 76 deaths (11.5%), and Group 2 had 158 deaths (10.8%), with no significant difference observed (P=0.575). Piecewise regression analysis revealed a correlation between serum-corrected CaPO_4_ levels and in-hospital mortality, identifying the lowest mortality probability at a CaPO_4_ level of 27 mg^2^/dL^2^ (Figure 3). After adjusting for potential confounders, an increase of 1 mg^2^/dL^2^ in corrected serum CaPO_4_ levels below 27 mg^2^/dL^2^ was associated with a 6.8% reduction in the risk of in-hospital mortality (OR: 0.932, 95% CI: 0.898-0.967). Conversely, for patients with CaPO_4_ levels ≥27 mg^2^/dL^2^, each 1 mg^2^/dL^2^ increase in CaPO_4_ significantly increased the risk of in-hospital mortality by 3.2% (OR: 1.048, 95% CI: 1.032-1.065) (Table 3).

**Table 3:** Piecewise regression analysis between admission-corrected serum CaPO4 and in-hospital mortality ^a^Adjusted for age, sex, race, underlying disease, the use of ACEI/ARB, diuretics, NSAID, aminoglycoside, macrolide, calcium supplement, vitamin D supplement, radiocontrast, vasopressor usage, and mechanical ventilator. The ORs presented are for the following parameters: CaPO_4_ ≤27 mg²/dL² and CaPO_4_ >27 mg²/dL². CaPO_4_, calcium phosphate; CI, confidence interval; OR, odd ratio; NSAID, non-steroidal anti-inflammatory drug; ACEI, angiotensin converting enzyme inhibitor; ARB, angiotensin receptor blocker

	Unadjusted			Adjusted^a^		
Variable	OR	95% CI	P-value	OR	95% CI	P-value
CaPO_4 _<27 mg^2^/dL^2^	0.919	0.887-0.953	<0.001	0.932	0.898-0.967	<0.001
CaPO_4_ ≥27 mg^2^/dL^2^	1.047	1.032-1.063	<0.001	1.048	1.032-1.065	<0.001

## Discussion

This study investigated the relationship between admission-corrected serum CaPO_4_ levels and the risks of in-hospital AKI and mortality, observing a bilinear pattern. Piecewise regression analysis revealed that for CaPO_4_ levels below 27 mg^2^/dL^2^, each one-unit increase was associated with a 6.0% decrease in AKI risk and a 6.8% decrease in mortality risk. Conversely, for CaPO_4_ levels at or above 27 mg^2^/dL^2^, each one-unit increase corresponded to a 4.8% increase in AKI risk (p<0.001) and a 3.2% increase in mortality risk (p<0.001). This marks a significant advancement over previous studies that utilized CaPO_4_ levels to determine associations with in-hospital AKI, where a linear association was typically assumed [9]. We identified 27 mg^2^/dL^2^ as the optimal cutoff point. By employing piecewise regression, which effectively captures this bilinear pattern, our analysis offers a more accurate description of the relationship.

We hypothesize that the observed relationship between elevated serum CaPO_4_ levels and AKI in this study can be explained by multiple mechanisms. An increased CaPO_4_ levels may lead to ischemic injury due to renal vasoconstriction, triggered by elevated serum calcium levels. Moreover, higher calcium levels could promote increased urinary calcium excretion, resulting from a compensatory increase in the filtered load and decreased tubular reabsorption [9,19]. Concurrently, hyperphosphatemia in distal tubules might induce acute phosphate nephropathy, a condition well-documented in previous studies [6-8].

Previous studies, primarily involving Caucasian cohorts, have established a U-shaped correlation between CaPO_4_ levels and mortality risk [19]. Our findings corroborate this pattern; however, our study, which primarily involves an Asian cohort, identifies a lower significant cutoff point for admission-corrected serum CaPO_4_ at >27 mg^2^/dL^2^ associated with increased in-hospital mortality, compared to ≥ 45 mg^2^/dL^2^ in prior studies. We hypothesize that these differences might be due to varying tubular capacities between races to handle CaPO_4_ excretion, underscoring the need to define race-specific cutoff values for CaPO_4_. The specific mechanisms by which elevated CaPO_4_ levels influence mortality outcomes remain unclear. However, it is possible that higher CaPO_4_ levels lead to complications such as AKI itself, which then adversely affects mortality outcomes [20].

Surprisingly, CaPO_4_ levels below 27 mg^2^/dL^2^ are associated with reduced mortality risks. This correlation might be explained by the likelihood of decreased development of AKI in this subgroup of patients, as previously discussed. However, it is important to note that low levels can lead to complications. Hypocalcemia is associated with cardiac arrhythmias, reversible cardiomyopathy, and heart failure. Similarly, hypophosphatemia can lead to conditions such as arrhythmias, myocardial dysfunction due to impaired energy metabolism, heart failure, hemolysis, rhabdomyolysis, encephalopathy, respiratory muscle weakness, and an increased need for inotropic support [21-27]. These complications may occur if the corrected CaPO_4_ levels are excessively low. Therefore, a critical question arises regarding the threshold at which lower CaPO_4_ levels shift from being beneficial to harmful. 

We acknowledge several limitations in our study. First, as a single-center study predominantly involving Asian patients, our results may not be generalizable to other populations. Further studies involving more diverse populations are necessary. Second, our inability to measure urine pH is a limitation, as alkaline urine can precipitate CaPO_4_ crystals, potentially leading to AKI. Additionally, the absence of data on the specific effects of the CaPO_4_ product on kidney tubules indicates that future studies should consider performing kidney biopsies to gain a deeper understanding of the underlying pathophysiology. Third, while serum parathyroid levels, vitamin D levels, and urine electrolytes are crucial for CaPO_4_ crystallization, our retrospective design limited the availability of these data. Fourth, the study may exhibit selection bias as it only included patients from the internal medicine department where serum calcium, phosphate, and albumin data were available. Fifth, although corrected serum CaPO_4_ is commonly used in clinical practice, we noted that albumin-corrected calcium concentration might overestimate hypercalcemia compared to ionized calcium, which, despite its higher cost and lesser availability in our setting, might be more accurate [28]. Finally, the retrospective cohort design of the study limits the ability to infer causality. Thus, randomized controlled trials are needed to better understand the causal relationships involving corrected CaPO_4_ levels, acute phosphate nephropathy, and AKI.

## Conclusions

Our study highlights a complex relationship between elevated serum CaPO_4_ levels and in-hospital outcomes, notably AKI and mortality, identifying a critical cutoff at CaPO_4_ >27 mg^2^/dL^2^. This bilinear association underscores the importance of close monitoring and management of serum CaPO_4_ levels in hospitalized patients to mitigate the risks associated with its elevation. The findings suggest that proactive management of these levels could significantly enhance patient outcomes by preventing complications associated with high CaPO_4_. Further studies are needed to confirm these results and develop effective clinical guidelines. 

## References

[1] Khwaja A (2012). KDIGO clinical practice guidelines for acute kidney injury. Nephron Clin Pract.

[2] Luyckx VA, Tonelli M, Stanifer JW (2018). The global burden of kidney disease and the sustainable development goals. Bull World Health Organ.

[3] Pan HC, Yang SY, Chiou TT (2022). Comparative accuracy of biomarkers for the prediction of hospital-acquired acute kidney injury: a systematic review and meta-analysis. Crit Care.

[4] Cartin-Ceba R, Kashiouris M, Plataki M, Kor DJ, Gajic O, Casey ET (2012). Risk factors for development of acute kidney injury in critically ill patients: a systematic review and meta-analysis of observational studies. Crit Care Res Pract.

[5] Gonlusen G, Akgun H, Ertan A, Olivero J, Truong LD (2006). Renal failure and nephrocalcinosis associated with oral sodium phosphate bowel cleansing: clinical patterns and renal biopsy findings. Arch Pathol Lab Med.

[6] Desmeules S, Bergeron MJ, Isenring P (2003). Acute phosphate nephropathy and renal failure. N Engl J Med.

[7] Connor A, Sykes L, Roberts IS, Weston CE (2008). Acute phosphate nephropathy after sodium phosphate preparations. BMJ.

[8] Asplin JR, Mandel NS, Coe FL (1996). Evidence of calcium phosphate supersaturation in the loop of Henle. Am J Physiol.

[9] Thongprayoon C, Cheungpasitporn W, Mao MA, Harrison AM, Erickson SB (2019). Elevated admission serum calcium phosphate product as an independent risk factor for acute kidney injury in hospitalized patients. Hosp Pract (1995).

[10] Block GA, Hulbert-Shearon TE, Levin NW, Port FK (1998). Association of serum phosphorus and calcium x phosphate product with mortality risk in chronic hemodialysis patients: a national study. Am J Kidney Dis.

[11] Cubbon RM, Thomas CH, Drozd M (2015). Calcium, phosphate and calcium phosphate product are markers of outcome in patients with chronic heart failure. J Nephrol.

[12] Ramírez-Morros A, Granado-Casas M, Alcubierre N (2017). Calcium phosphate product is associated with subclinical carotid atherosclerosis in type 2 diabetes. J Diabetes Res.

[13] Zuk A, Bonventre JV (2016). Acute kidney injury. Annu Rev Med.

[14] Cheng X, Wu B, Liu Y, Mao H, Xing C (2017). Incidence and diagnosis of acute kidney injury in hospitalized adult patients: a retrospective observational study in a tertiary teaching Hospital in Southeast China. BMC Nephrol.

[15] Parent X, Spielmann C, Hanser AM (2009). ["Corrected" calcium: calcium status underestimation in non-hypoalbuminemic patients and in hypercalcemic patients]. Ann Biol Clin (Paris).

[16] Payne RB, Little AJ, Williams RB, Milner JR (1973). Interpretation of serum calcium in patients with abnormal serum proteins. Br Med J.

[17] Levey AS, Stevens LA, Schmid CH (2009). A new equation to estimate glomerular filtration rate. Ann Intern Med.

[18] Charlson M, Szatrowski TP, Peterson J (1994). Validation of a combined comorbidity index. J Clin Epidemiol.

[19] Thongprayoon C, Cheungpasitporn W, Mao MA, Erickson SB (2020). Calcium-phosphate product and its impact on mortality in hospitalized patients. Nephrology (Carlton).

[20] Coca SG, Yusuf B, Shlipak MG, Garg AX, Parikh CR (2009). Long-term risk of mortality and other adverse outcomes after acute kidney injury: a systematic review and meta-analysis. Am J Kidney Dis.

[21] Suzuki T, Ikeda U, Fujikawa H, Saito K, Shimada K (1998). Hypocalcemic heart failure: a reversible form of heart muscle disease. Clin Cardiol.

[22] Hurley K, Baggs D (2005). Hypocalcemic cardiac failure in the emergency department. J Emerg Med.

[23] Charniot JC, Alexeeva A, Laurent S (2001). [Reversible hypokinetic cardiomyopathy revealing severe hypocalcemia]. Arch Mal Coeur Vaiss.

[24] Wong CK, Pun KK, Cheng CH, Lau CP, Leung WH, Chan MK, Yeung DW (1990). Hypocalcemic heart failure in end-stage renal disease. Am J Nephrol.

[25] Miura S, Yoshihisa A, Takiguchi M (2015). Association of hypocalcemia with mortality in hospitalized patients with heart failure and chronic kidney disease. J Card Fail.

[26] Geerse DA, Bindels AJ, Kuiper MA, Roos AN, Spronk PE, Schultz MJ (2010). Treatment of hypophosphatemia in the intensive care unit: a review. Crit Care.

[27] Halevy J, Bulvik S (1988). Severe hypophosphatemia in hospitalized patients. Arch Intern Med.

[28] Gøransson LG, Skadberg Ø, Bergrem H (2005). Albumin-corrected or ionized calcium in renal failure? What to measure?. Nephrol Dial Transplant.

